# Calculi of the Nasal Fossæ

**Published:** 1846-03

**Authors:** M. Demarquay


### Collectanea.

*Calculi of the Nasal Fossæ,* by M. DEMARQUAY.—Calculi of the nasal fossæ, which Graaf calls rhinolithes, appear to have been first mentioned in 1502, by Joseph Mathias de Gardi. Cases of this disease have subsequently been given by Thomas Bartholin, 1654; Clander, 1685; Kern, 1700; Vitus Reidlinus, 1706; Wepfer, 1727; Ruysch, 1733; Plater, 1736; Horn, 1788; Saviales, 1814; Graff, 1828; Mr. Thouret, 1829; and Sir B. Brodie, (*Lancet,* July 6,) 1844. The cases quoted by these authors, M. Demarquay gives at length, and founds on them the following description of the disease:

Nasal calculi may exist alone, or in variable numbers. They may develope themselves on either side, and in the inferior or the superior regions of the nasal fossæ. It is, however, more especially in the inferior meatus that they appear to originate. They may be found in the frontal sinuses, or even in the maxillary sinus, and thence pass into the nasal fossæ. They may completely obstruct the cavities of the nose, incline the septum to one side, or even destroy it. Their volume varies from that of a pea, to that of a pigeon's egg; their color is black, grey, or white; their surface is uneven, and their centre is often constituted by a foreign body, or by the root of an incisor tooth. They are formed of the elements which are found in the secretions of the nasal fossæ, and in the tears, viz. mucus, phosphate of lime, and the carbonates of lime and magnesia.

The causes which give rise to nasal calculi are obscure. Graaf attributes them to gout, but his own case is the only one in which the gouty diathesis existed. Chronic inflammation of the nasal fossæ, and of the lachrymal gland, appear the most probable causes of this affection. In many cases, the calculus appears to have formed round a foreign body. A cherry-stone, for instance, the root of a tooth, or some other substance. The presence of one or more calculi in the nasal fossæ occasions so little annoyance in some as to be scarcely perceived, whilst in other cases the symptoms may be sufficiently severe to necessitate surgical interference. The most frequent symptoms are, a certain degree of dryness in the affected nostril, accompanied by a sensation of obtusion and weight, and by difficulty of respiration. Sometimes there is acute pain in the nose or forehead, of either a constant or intermitting nature. The inflammation of the surrounding parts may become severe, and give rise to an abundant fœtid suppuration. The nose may become externally deformed. The eye may participate in the inflammation, or be bathed with tears, as in fistula lachrymalis. This is more especially the case when the calculi form in the inferior meatus. On dilating the nostrils, the foreign body is to be recognized. When this occurs a metallic sound, or the polypus forceps, should be introduced. The characteristic sound produced by their striking against the calculus will at once show what is the disease. If situated in the frontal sinuses, or very high in the nasal fossæ, they may not be recognizable by either of these modes of exploration. Calculi thus developed have often remained very long without being recognized. Sometimes they have been expelled in a fit of coughing or sneezing, but they have generally been extracted by the hand of the surgeon. Nasal calculus has given rise to numerous errors of diagnosis, the symptoms which it produces have been attributed to ozæna, to disease of the bones of the nose, &c. Generally speaking, however, it is not difficult for a surgeon, who is aware of the existence of such a disease, to recognize its presence.

The first indication to fulfil in the treatment is the extraction of the calculus, an operation which it is not always easy to accomplish. The extraction may generally be effected with a pair of polypus forceps. It must, however, be done with care, owing to the inequality of surface which the calculi present. When the calculi have been removed, the surgeon must, by an appropriate treatment, combat the inflammatory symptoms to which they have given rise. Emollient and astringent injections are often very useful. If it is supposed that the presence of the calculi is connected with any general diathesis, this must be treated by appropriate remedies.—*Half-yearly Abstract of Med. Sci.*

